# Route to Topological Superconductivity via Magnetic Field Rotation

**DOI:** 10.1038/srep15302

**Published:** 2015-10-19

**Authors:** Florian Loder, Arno P. Kampf, Thilo Kopp

**Affiliations:** 1 Center for Electronic Correlations and Magnetism, Experimental Physics VI, Institute of Physics, University of Augsburg, 86135 Augsburg, Germany; 2 Center for Electronic Correlations and Magnetism, Theoretical Physics III, Institute of Physics, University of Augsburg, 86135 Augsburg, Germany

## Abstract

The verification of topological superconductivity has become a major experimental challenge. Apart from the very few spin-triplet superconductors with *p*-wave pairing symmetry, another candidate system is a conventional, two-dimensional (2D) *s*-wave superconductor in a magnetic field with a sufficiently strong Rashba spin-orbit coupling. Typically, the required magnetic field to convert the superconductor into a topologically non-trivial state is however by far larger than the upper critical field *H*_c2_, which excludes its realization. In this article, we argue that this problem can be overcome by rotating the magnetic field into the superconducting plane. We explore the character of the superconducting state upon changing the strength and the orientation of the magnetic field and show that a topological state, established for a sufficiently strong out-of-plane magnetic field, indeed extends to an in-plane field orientation. We present a three-band model applicable to the superconducting interface between LaAlO_3_ and SrTiO_3_, which should fulfil the necessary conditions to realize a topological superconductor.

While topologically non-trivial superconducting (SC) states have been established theoretically in numerous systems^1^,^2^,^3^,^4^,^5^,^6^, an experimental verification of such a state is still awaited. This is largely a consequence of the required conditions, which tend to counteract superconductivity itself. A topologically non-trivial state is generally described by a non-zero momentum space Berry phase *γ* = 2*πC* with an integer *C* whenever there is an energy gap separating occupied from unoccupied states^1^. The superconducting state can acquire a finite Berry phase through a chiral order parameter, and also via gapping a chiral normal-metal state upon entering a conventional SC state. Examples of the former case are selected spin-triplet states, e.g., the A-phase of superfluid ^3^He 1 and most likely the superconducting phase of Sr_2_RuO_4_^7^,^8^. Very recently, a similar topological character was also proposed for the superconducting state in strongly underdoped cuprates^9^ in which a gap exists even along the nodal direction of a *d*-wave order parameter^10^. These proposals are built on states of matter, where the topological nature is an intrinsic property. The chiral order parameter however requires a very special pairing interaction; the *p*-wave states are rare in nature and pose considerable experimental challenges.

On the other hand, if the material provides a chiral band structure by itself, a conventional BCS superconductor with an *s*-wave order parameter can be topologically non-trivial as well. Most often discussed is an *s*-wave superconductor with a Rashba type spin-orbit coupling (SOC) in two dimensions^5^. On the two Fermi surface sheets generated by Rashba SOC the electron spins wind around in opposite directions (see Fig. 1 or e.g. ref. 11). Therefore, in order to reach a state with a finite overall Berry phase, an additional Zeeman field is needed which is strong enough to depopulate one of the SOC split bands. The topological character of the resulting SC state is equivalent to the quantum-Hall state. Such states are classified by a topological invariant, the so-called TKNN integer *C* (after Thouless, Kohmoto, Nightingale, and Nijs)^12^. If the magnetic field is perpendicular to the plane of the 2D superconductor, the minimal Zeeman splitting required to reach the topological phase is 
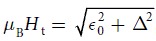
, where 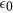 measures the distance of the band energy at **k** = 0 to the Fermi energy, and Δ is the SC energy gap^5^. The obstacle for realizing this topological state experimentally is to find a system which remains superconducting in the required high magnetic fields. Suggested model systems are, e.g., neutral ultra-cold atoms in an optical trap^5^, or heterostructures where Cooper pairs are induced through the proximity effect^2^,^13^,^14^,^15^,^16^.

The problem of realizing the topological *s*-wave state has two distinct aspects: (*i*) *μ*_B_*H*_t_ must be larger than Δ. While the presence of the Rashba SOC allows in principle *s*-wave superconductivity in a Zeeman field larger than Δ, the orbital critical field *H*_c2_ is typically much smaller. (*ii*) The superconductor must have *ε*_0_ smaller than the Zeeman splitting. This requires a low band filling and, therefore, superconductivity must be stabilized by yet another band with larger filling. In this article, we address both of these aspects and demonstrate that the problems can be overcome in real solid-state systems.

A simple way to circumvent the orbital critical field *H*_c2_ is to rotate the magnetic field into the plane of the 2D superconductor. The in-plane field however leads to an unusual type of pairing. In the presence of Rashba SOC, an in-plane magnetic field shifts the Fermi surfaces out of the Brillouin-zone center (cf. Fig. 1), and the electron pairs thereby acquire a finite center-of-mass momentum (COMM)^11^. Edge states in an in-plane magnetic field have recently been investigated for *p*-wave superconductors, but with zero COMM^4^,^17^. As we show here, the inclusion of a finite COMM in such a field geometry is indispensable for the discussion of topology. Specifically, we analyze the topological properties of an *s*-wave superconductor under rotation of the magnetic field within a fully self-consistent treatment of the SC order parameter. It is verified that the topological state reached in out-of-plane fields indeed persists to in-plane field orientations, if the COMM is appropriately chosen to minimize the free energy. For in-plane fields the energy gap closes, accompanied by a topological transition. Nevertheless, chiral edge modes remain even for a regime with a closed gap.

We discuss the experimental realizability of a topological *s*-wave superconductor in a nearly in-plane magnetic field. As a candidate system, which can possibly fulfil the required conditions, we consider the metallic LaAlO_3_-SrTiO_3_ (LAO-STO) interface^18^,^19^. For this system, several models are proposed for a topologically non-trivial superconducting state, which rely on an unconventional order parameter^20^,^21^,^22^. Assuming instead an *s*-wave pairing state, we demonstrate that a multi-band model involving the titanium *t*_2*g*_ orbitals allows for a topologically non-trivial superconductor in a realistic parameter regime for the LAO-STO interface. We suggest that it may be achieved with the currently used experimental setups.

## Results

In order to investigate the magnetic-field dependence of an *s*-wave superconductor with Rashba SOC in transparently simple terms, we use a one-band tight-binding model on a square lattice in the *x*-*y*-plane at zero temperature. In our analysis of the topology upon rotating the magnetic field **H** into the plane, we include the Zeeman coupling of the electrons to the magnetic field, but neglect the orbital coupling. This approximation is well justified for the nearly in-plane field orientation on which we focus here; but orbital effects are necessarily important for the superconducting state in an out-of-plane magnetic field.

**Figure 1:**
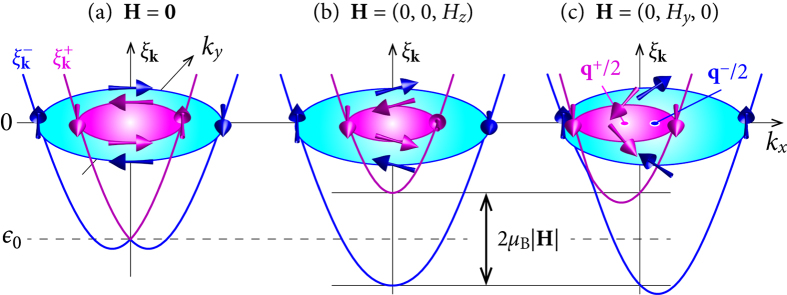
Energy bands 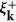 (pink) and 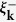 (blue) and Fermi surfaces with *α* > 0 and magnetic filed |H| < *H*_t_. (**a**) For **H** = 0, the two bands touch at **k** = 0. (**b**,**c**) For |**H**| > 0, the band splitting at **k** = 0 is equal to the Zeeman splitting 2*μ*_B_|**H**|. The centers of the shifted Fermi surfaces in (**c**) are at the momenta momenta **q**^+^/2 = (*q*^+^/2, 0) and *q*^−^/2 = (*q*^−^/2, 0), respectively. Although *q*^+^ ≈ −*q*^−^, their absolute values are in general different. In (**c**), the spins on the *k*_*x*_-axis orient according to the magnetic field rather than according to the SOC, if *H*_*y*_ > *α*|sin *k*_F_|. Note that the Fermi energy is somewhat larger than |*ε*_0_| because of the SOC induced band splitting.

The Rashba SOC and the Zeeman coupling to the magnetic field **H** are combined into


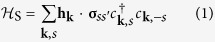


with *s* = ±1, the Bloch vector **h**_**k**_ = *α***g**_**k**_ + *μ*_B_**H**, and **g**_**k**_ = (sin *k*_*y*_, −sin *k*_*x*_, 0); ***σ*** is the vector with the Pauli matrices as components. The strength of the Rashba SOC *α* derives originally from the Dirac Hamiltonian, but may have other sources in multi-band systems (see section “Discussion”). Diagonalizing the kinetic energy together with 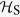 gives the two chiral energy bands 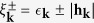, where 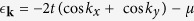 with the nearest-neighbor hopping amplitude *t* and the chemical potential *μ* (thus 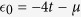). In these bands, the spin is either parallel or antiparallel to **h**_**k**_ and has a component which rotates either counter-clockwise or clockwise upon circulating the Fermi surfaces (see Fig. 1).

### Out-of-plane magnetic field

For an out-of-plane magnetic field with *H*_*x*_ = *H*_*y*_ = 0, the topological properties of the superconducting state are readily established (see e.g. Ref. 5). Its Hamiltonian 
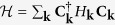
 is represented by the 4 × 4 matrix


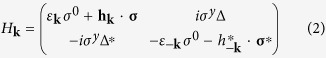


with 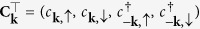 and *σ*^0^ is the 2 × 2 unit matrix; the pairing field Δ is calculated self-consistently from Eq. (7) (with **q** = 0). The four eigenenergies *E*_**k**,*n*_ obtained from diagonalizing (2) are generally the solutions of a 4th order polynomial, but simplify to


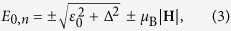


for *k*_*x*_ = *k*_*y*_ = 0, since **g**_**k**=0_ = 0. The number *n* labels the four combinations of the plus and minus signs. It follows that the energy gap closes at **k** = 0 for 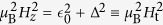, which thereby allows for a topological transition^5^,^6^.

The topological character of the SC state is given by the TKNN number


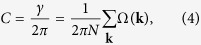


where


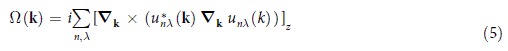


is the *z*-component of the Berry curvature^12^. The sum over *n* runs over the occupied bands *E*_**k**,*n*_ < 0 and *λ* = 1, …, 4 labels the components of the eigenvectors **u**_*n*_(**k**) of the matrix *H*_**k**_. The number *C* is integer valued, if the occupied energy levels are separated by a finite gap from the unoccupied levels. The value of *C* and therefore the topology of the quantum state changes when the energy gap closes at |*H*_*z*_| = *H*_t_. For magnetic fields |*H*_*z*_| > *H*_t_, the energy gap opens again. This reopening of a gap, above the paramagnetic limiting field *μ*_B_|*H*_*z*_| = Δ, is tied to the presence of SOC, which protects the spin-singlet pairing channel. (In the presence of SOC, the spin susceptibility *χ*_S_ of the spin-singlet superconductor remains finite down to *T* = 0. In particular, if Δ ≪ *α*, *χ*_S_ is almost equal the Pauli susceptibility of the normal state. Therefore, the Clogston-Chandrasekhar paramagnetic limit 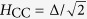 23 does also not apply.)

**Figure 2:**
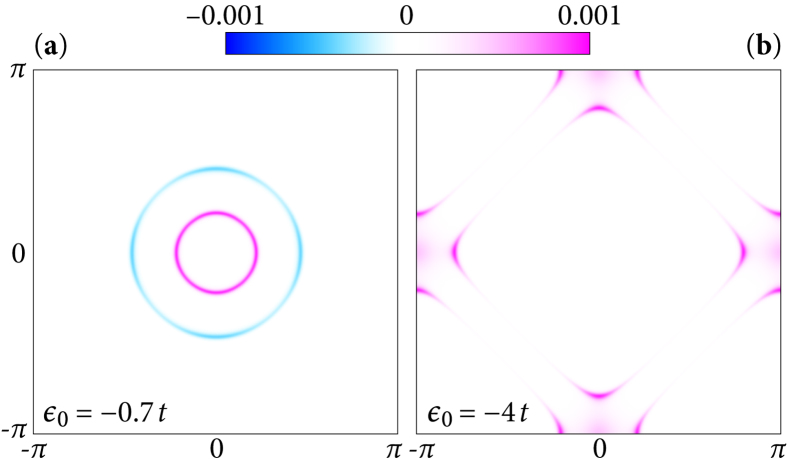
*z*-component of the Berry curvature Ω(**k**) for an out-of-plane magnetic field *H*_*z*_ in the SC state. Ω(**k**) is finite within the window Δ below the Fermi energy. (**a**) In the topologically trivial state, (here: 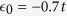, 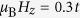 and Δ is fixed to 0.1 *t*), the total Berry curvature integrates to zero over the Brillouin zone. (**b**) In the topological situation (B) (see main text, 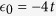 and 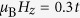), the Berry curvature integrates to 2*πC* = 4*π* over the Brillouin zone.

Only the momenta **k** for which 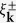 lie within a window Δ below the Fermi energy contribute to *C*. The sign of this contribution reflects the winding direction of the *x*–*y* components of the spin 
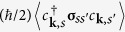
 in momentum space (see Fig. 1). If |*H*_*z*_| < *H*_t_, the **k**-integrated Berry curvatures in the vicinity of the two normal-state Fermi surfaces cancel exactly [Fig. 2(a)] and consequently *C* = 0.

The topological state emerging for |*H*_*z*_| > *H*_t_ is of different nature in two distinct density regimes:

1. small electron density (*μ* < −2*t*): the condition |*H*_*z*_| > *H*_t_ leads to 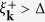 for all **k** and therefore the 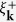-band is empty and does not contribute, i.e., the pink (positive) contributions to *C* in Fig. 2(a) vanish. Consequently, the superconducting state is characterized by 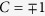, depending on the sign of *H*_*z*_. This situation is realized for small band fillings.
2. densities near half-filling (|*μ*| < 2*t*): in this regime, two separate topological transitions are possible. At a magnetic field 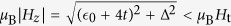, the character of the 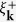-band changes from particle- to hole-like and thereby reverses the sign of Ω(**k**) in the vicinity of the corresponding Fermi surface [Fig. 2(b)]. Therefore, a topological transition to *C* = ±2 occurs, depending on the sign of *H*_*z*_, with both, the 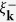
- and the 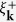-band, partially occupied. A realization of this superconducting state close to half filling is however unlikely due to possibly competing orders. For an even larger magnetic field *μ*_B_|*H*_*z*_| > *H*_t_, the 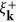-band is again lost, and *C* changes to  = ±1. The topological properties of both cases, (A) and (B), correspond to those described in ref. 4 in the context of spin-triplet superconductors.

A special characteristic feature of *s*-wave superconductivity in the presence of Rashba SOC is that the magnetic field induces an inter-band pairing contribution where a quasi-particle of the 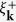-band is paired with one from the 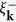-band at opposite momentum. This pairing contribution induces interior energy gaps above and below the Fermi energy (clearly visible e.g. in the spectra presented below, cf. ref. 24). A more detailed discussion of the relation between intra- and inter-band pairing is given in the Supplementary Informations.

### In-plane magnetic field

For a finite in-plane magnetic-field component 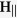, the Fermi surfaces are shifted out of the Brillouin-zone center in opposite directions perpendicular to 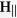 [Fig. 1(c)], since 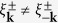. The pairing of electrons with momenta **k** and −**k** is thereby suppressed. Instead, pairs are formed in which electrons have momenta **k** and −**k** + **q**^±^, respectively, where the COMMs **q**^±^ account for the Fermi surface shifts^11^,^25^,^26^,^27^ (see Supplementary Informations, Sec. B).

The SC ground state with an in-plane magnetic field component therefore contains in general two order parameters 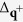 and 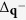. These enter the generalized on-site pairing term as


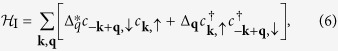


where **q** = **q**^+^, **q**^−^ 28. The singlet order parameter for COMM **q** is calculated self-consistently from


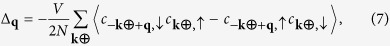


where *V* is the strength of the pairing-interaction. With increasing in-plane magnetic-field strength 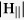, the difference |**q**^+^ − **q**^−^| grows. Such a finite COMM state is spatially non-uniform^28^ with lines of zero pair density, similar to the SC state proposed by Larkin and Ovchinnikov for a singlet superconductor in a strong Zeeman field^29^. Characteristic for this state is a mixing of intra- and inter-band pairing and the absence of a full energy gap (see Supplementary Informations)^11^,^28^.

For 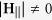, the topological characterization is more involved. In situation (B), close to half-filling, both bands 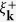 and 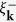 are partially occupied and consequently two COMMs **q**^±^ appear. Therefore no full energy gap is present, which implies that C is not an integer and therefore unsuitable to characterize the topology (nevertheless, edge modes may still occur, see Supplementary Informations). For this reason, we focus below on situation (A) when only the lower band 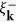 is occupied and therefore only electron pairs with COMM **q**^−^ form. Such a state is spatially uniform and similar to the state introduced by Fulde and Ferrell^30^, but it carries a finite charge current perpendicular to 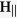. If also *H*_*z*_ ≠ 0, it exhibits a full energy gap and therefore *C* is integer-valued, except for a limited crossover region 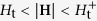 discussed below. Equation (3) and therefore the definition of *H*_t_ is valid as well in the presence of an in-plane magnetic field component whereby *H*_*z*_ is replaced by |**H**|.

If |**H**| > *H*_t_, one finds that *α*|**g**_**k**_| < *μ*_B_|**H**| for all occupied momenta **k** in the 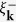-band. Furthermore, if **H** is strictly in-plane, say along the *y*-axis, **h**_**k**_ is parallel to **H** on the *k*_*x*_-axis and therefore the spins are parallel (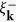-band) or anti-parallel (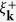-band) to **H** as well [Fig. 1(c)]. As a consequence, no intra-band pairing in the singlet channel is possible for *k*_*y*_ = 0, i.e., the intra-band energy gap closes at the two Fermi points with *k*_*y*_ = 0. This gap closing for an in-plane field orientation corresponds to a topological transition from *C* = −1 to *C* = 1.

The topological phase of situation (A) with an in-plane magnetic field component is described by the same Hamiltonian as in Eq. (2) replacing Δ by 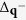. In the following we show that the topological state found for a sufficiently strong out-of-plane magnetic field can persist when the field is rotated — even down to an in-plane field orientation with arbitrarily small *H*_*z*_.

### Phase diagram and edge states

**Figure 3:**
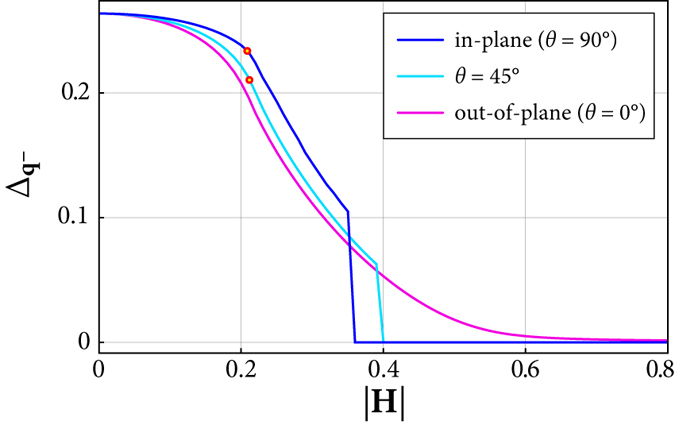
Self-consistent solutions of the SC order parameter 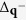 for three magnetic-field directions and *V* = 4 *t*, *α* = 0.5 *t* and a constant electron density *n* = 0.05. This value of *n* corresponds to 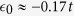. For such low densities, a large interaction strength *V* is required to obtain a reasonably large order parameter. For each value of |**H**|, **q**^−^ = (*q*, 0) is obtained by minimizing the free energy. The red circles indicate the magnetic field strength above which a finite COMM *q* ≠ 0 is present.

We start the analysis of the SC state with the discussion of the self-consistent solutions of the SC order parameter. Figure 3 shows the magnetic-field dependence of 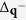 for different angles *θ* of the field direction. The Rashba SOC ensures the presence of a finite in-plane spin component which allows for singlet pairing. Therefore the Zeeman coupling to a field in *z*-direction (*θ* = 0° and **q**^−^ = 0) cannot wipe out superconductivity completely (pink curve) when orbital depairing is not included. A finite in-plane field component leads instead to a finite critical magnetic field *H*_c_(*θ*), above which there are no solutions for 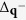.

**Figure 4:**
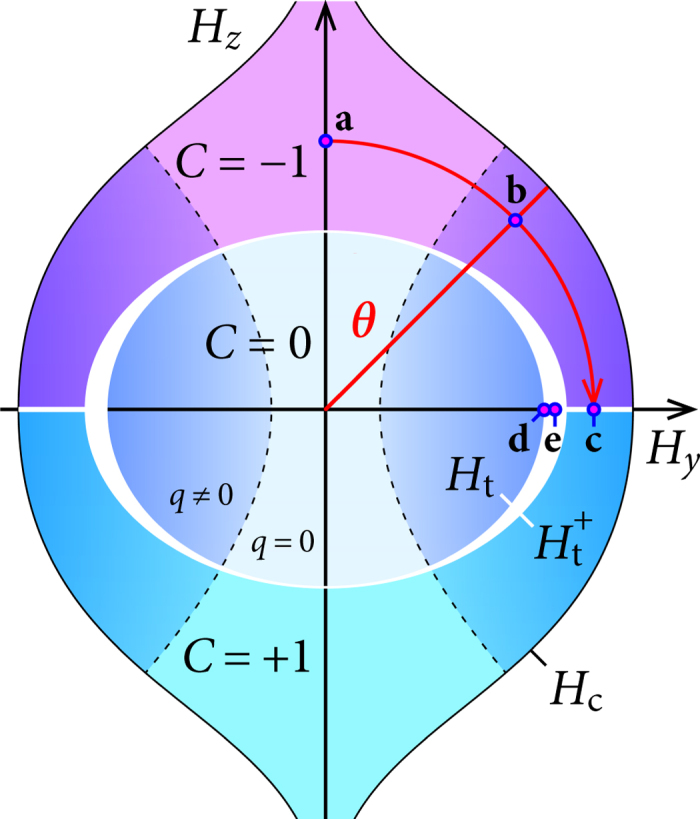
Phase diagram showing the topologically different SC states as a function of out-of-plane magnetic field *H*_*z*_ and in-plane magnetic field *H*_*y*_, not including orbital coupling to the magnetic field. The blue circles (a–e) mark the *H*_*y*_ − *H*_*z*_-points for which the energy spectra are shown in Fig. 5. The dashed lines indicate the transition from zero COMM to finite COMM pairing.

An interesting result is the somewhat larger value for Δ_**q**_ in an in-plane magnetic field 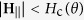 than in an out-of-plane magnetic field of the same magnitude. Consequently, the magnetic field *H*_t_(*θ*) at which the energy gap closes, grows with increasing angle *θ* and is maximal for an in-plane direction. Likewise, the field 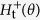 above which the energy gap opens again and the topological state emerges, is maximal for *θ* = 90°, whereas 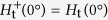. The resulting phase diagram for different magnetic-field orientations is qualitatively drawn in Fig. 4. The topologically trivial SC state (*C* = 0) is bounded by the ellipse given by *H*_t_(*θ*), which itself is within the slightly larger ellipse formed by 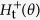. The white regime in between separates the state with *C* = 0 from the states with *C* = ±1. In this crossover region the energy gap is closed and *C* is not an integer. A further topological transition occurs for the in-plane field orientation *θ* = 90° and 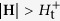: if *θ* sweeps through 90°, the out-of-plane field component *H*_*z*_ changes sign and, accordingly, *C* changes from −1 to 1. As discussed above, the energy gap is closed as well along this transition line.

**Figure 5:**
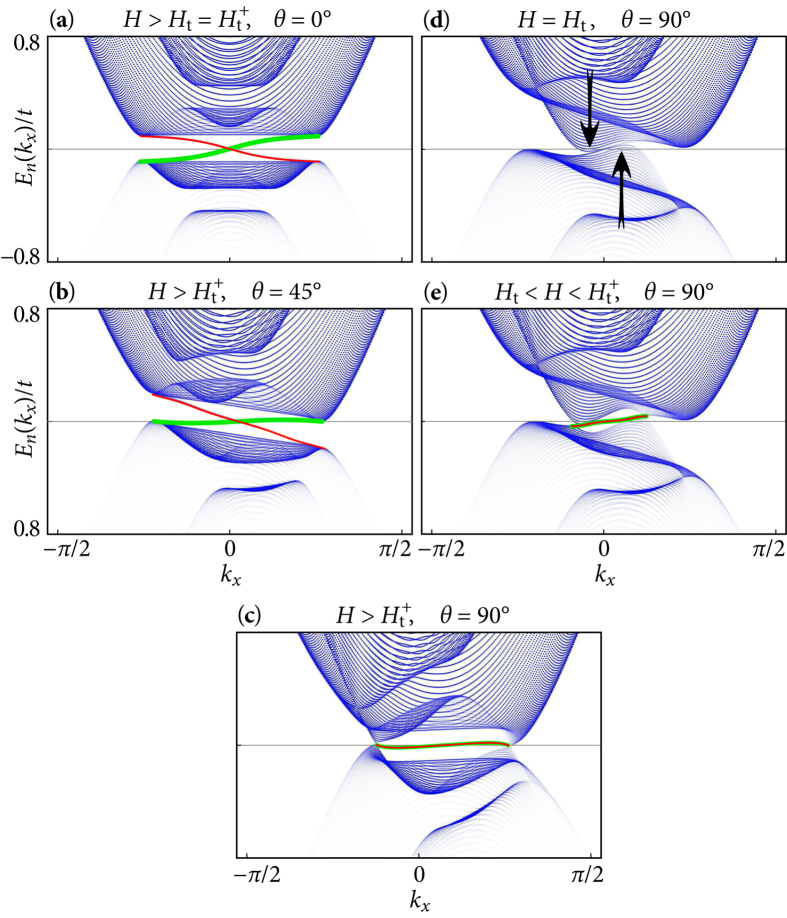
Energy spectra *E*_*n*_(*k*_*x*_) for a stripe geometry with 600 × 100 sites, open boundary conditions and in-plane magnetic field component in *y*-direction, and parameters *V*, *α*, and *n* as in Fig. 3. (**a**–**c**) The evolution of the edge modes (green line: upper edge, red line: lower edge) upon rotating the magnetic field is shown for (**a**) 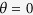, (**b**) 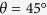, (**c**) 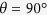, and 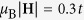. The self-consistently calculated order parameters 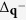 and COMMs **q**^−^ are (**a**) 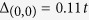, (**b**) 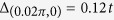, and (**c**) 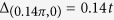. (**d**,**e**) illustrate the crossover regime 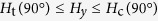: (**d**) 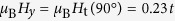 and 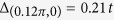, and (**e**) 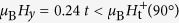 and 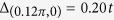. The black arrows in (**d**) indicate the partial occupation of states originating from the 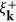-band. The opacity of each point encodes the weight with which the corresponding state contributes to the density of states.

The importance of finite-COMM pairing for the topological properties of the SC state is illustrated using the energy spectra shown in Fig. 5. These spectra are calculated for a stripe geometry with open boundary conditions in *y*-direction, which allows for in-gap edge modes (drawn in red and green). We choose the in-plane magnetic-field component in the *y*-direction and thereby obtain a shift of the Fermi surfaces out of the Brillouin-zone center in *k*_*x*_-direction. Therefore a COMM **q**^−^ = (*q*, 0) (*q* ≥ 0 for *H*_*y*_ > 0) has to be taken into account for pairing in the 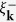-band. The free energy of the SC state is minimized for the smallest *q* which is still large enough to avoid an indirect closing of the energy gap (see Fig. 5(a–c) and Sec. B of the Supplementary Informations). The TKNN number *C* thereby remains well defined up to the magnetic-field direction *θ* = 90°. For *θ* → 90° [Fig. 5(c)], the energy gap closes at two *k*_*x*_-points, and a topological transition occurs. The magnetic field strength, above which a finite **q**^−^ is required (dashed line in Fig. 4), depends on the angle *θ*. In the limit *θ* → 90°, it is necessarily smaller than *H*_t_ and approaches *H*_t_ for vanishing SOC. For every change in the field orientation the COMM **q**^−^ has to be recalculated self-consistently. Since the calculations are performed on a finite lattice, **q**^−^ evolves necessarily in discrete steps, determined by the system size, upon sweeping the angle *θ*. Up to this unavoidable discreteness, the onset of a finite **q**^−^ is a smooth transition, across which the energy gap evolves continuously.

Eventually, Fig. 5 also shows the evolution of the in-gap edge modes (green and red lines) under the rotation of the magnetic field. (Note that for *θ* > 0, the direction of the in-plane field component relative to the boundary is important. If the in-plane field is orthogonal to the boundary (here: *y*-direction), the *k*_*x*_-dispersion of the edge mode is unperturbed and remains gapless. If there is a field component parallel to the boundary (in *x*-direction), the edge-mode disperses in *y*-direction as well. Since the dispersion in *y*-direction is quantized through the edges, the edge modes acquire an energy gap around the Fermi energy (also observed for the edge modes in chiral *p*-wave superconductors^4^,^17^). This mesoscopic energy gap is of the size of the normal-state level spacing around *E*_F_ and vanishes like 1/*M* in the thermodynamic limit, where *M* is the number of lattice sites in *y*-direction.) Starting from *θ* = 0°, the energy difference 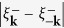 grows for increasing *θ* and thus the dispersion of the two opposite edge modes becomes asymmetric [Fig. 5(b)]. Upon approaching *θ* = 90°, the energy gap closes at two *k*_*x*_ points [Fig. 5(c)]. Consequently, *q* must be chosen to ensure that these closing points are located at the Fermi energy in order to prevent the energy bands above and below from overlapping. In this situation, the two edge modes are degenerate. Indeed, the two modes carry edge currents flowing in the same direction opposite to the flow of the bulk current. These modes are similar to the edge modes found for *p*-wave superconductors in an in-plane magnetic field^17^,^31^,^32^, except for the presence of a finite COMM pairing due to the Rashba SOC.

Figure 5(d,e) illustrate the gap closing and the emergence of edge modes in the regime 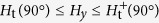. At *H*_*y*_ = *H*_t_(90°), the energy gap closes at *k*_*x*_ = 0 [Fig. 5(d)]. However, the minimum of the 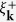-band is at a momentum *k*_*x*_ < 0 and somewhat below the Fermi energy (indicated by the left black arrow), whereas the maximum of its mirrored hole-band is at a momentum *k*_*x*_ > 0 somewhat above the Fermi energy (right black arrow). Thus, the 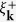-band and its mirrored hole band 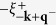 overlap indirectly. Superconductivity nevertheless persists because of the gain of condensation energy from the 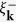-band. In the regime 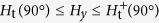, a direct gap opens again around *k*_*x*_ = 0 with two gap-crossing edge modes, although they are no longer protected by topology [Fig. 5(e)].

For magnetic fields 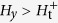, all states of the 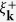-band are above the Fermi energy. In this regime an infinitesimally small out-of-plane magnetic-field component *H*_*z*_ is sufficient to remove the two gap-closing points and ensure well defined TKNN numbers *C* = ±1. Eventually, superconductivity breaks down at *H*_*y*_ = *H*_c_(*θ*): Above this critical magnetic field, the two gap-closing points move into the continuum of the energy bands above and below the Fermi energy. The upper and lower bands therefore overlap and the self-consistent solution for the SC order parameter is lost. Although 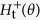 depends only weakly on the SOC strength *α*, the critical magnetic field *H*_c_(*θ*) grows with increasing *α*. In order to obtain 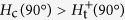, it is required that *α* > *μ*_B_*H*_*y*_.

## Discussion

How can the above topologically non-trivial SC state be realized in a solid-state system? We infer that an ideal candidate system would consist of several partially filled energy bands with a sizable Rashba SOC. Such a model^33^,^34^ was proposed e.g. to describe the physics of the conducting interface between LaAlO_3_ and SrTiO_3_^18^,^19^.

The condition 
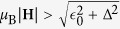
 for *C* ≠ 0 implies that the lower limit for the Zeeman splitting is given by Δ. The corresponding magnetic field is typically larger than the upper critical field *H*_c2_ above which orbital depairing destroys superconductivity. Therefore the topological state is not accessible with a magnetic field oriented along the *z*-axis. The topological state can be reached only for a nearly in-plane field orientation with *H*_*z*_ < *H*_c2_ but 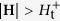. This excludes the situation (B) with a close to half-filled band, because of the presence of two different COMMs **q**^±^ as discussed above. The alternative situation, on the other hand, requires that 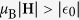, which is close to the Fermi energy *E*_F_ in a one-band model. For a band filling large enough to allow for a SC state (for a reasonable interaction strength *V*), *E*_F_ for this partially filled band must be at least several meV. The magnetic field required to overcome this energy would be far too large for experimental realizations.

**Figure 6:**
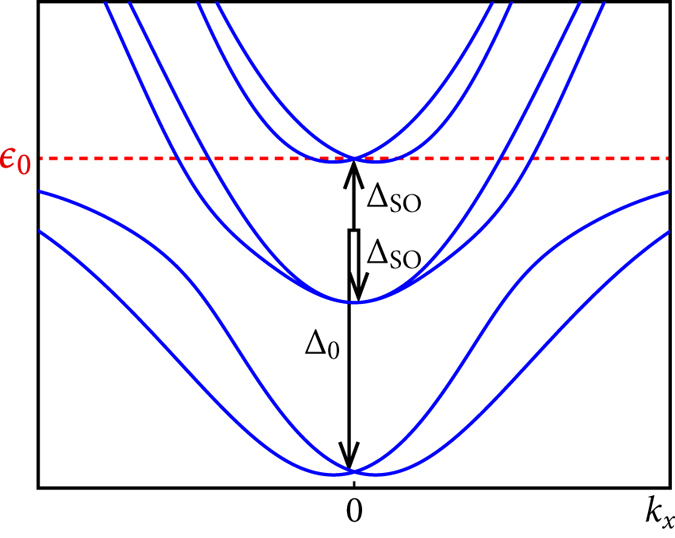
Band structure of the three-band model for *k*_*y*_ = 0. In order to ensure 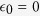 (red dashed line), the Fermi energy should be at the degeneracy point of the upper, Rashba-like doublet. The parameters are here: 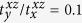, Δ_0_ = *t*, 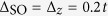.

In a multi-band setup, 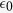 refers to the energy of the degeneracy point of a spin-orbit coupled doublet hosting the possibly topological state (see Fig. 6), relative to *E*_F_. The necessity of stabilizing a superconducting state with 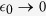 requires the presence of at least two bands at *E*_F_: A lower band provides the electron density for a sufficient gain of condensation energy in the superconducting state, whereas a second band has a minimum close enough to the Fermi energy so that it can be emptied or partially filled through an external control parameter.

A model which implements these features, and additionally also a strong Rashba-like SOC, recently emerged from the theoretical description of the LAO-STO interface. At this interface, the intrinsic electrostatic potential in LAO induces a nearly 2D electron liquid, which resides mainly in the titanium 3*d t*_2*g*_ orbitals of the first TiO_2_ layer^19^,^35^. The band-structure of this oxide interface provides a prototype for a class of similar interface systems that we introduce here by constructing a tight-binding Hamiltonian 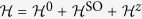 for the three *t*_2*g*_ bands *d*_*xy*_, *d*_*xz*_, and *d*_*yz*_, following refs 33,34. The free kinetic energy is given by


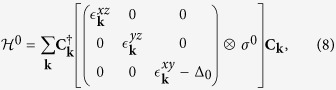


where *σ*^0^ is the 2 × 2 unity matrix and 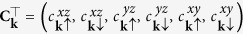. The hopping matrix elements 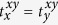 for the *d*_*xy*_ band are identical in the *x*- and *y*-direction, whereas they are different for the *d*_*xz*_ and *d*_*yz*_ bands: 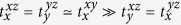. Furthermore, the in-plane *d*_*xy*_ orbital is lowered in energy by Δ_0_ relative to the out-of-plane *d*_*xz*_ and *d*_*yz*_ orbitals because of the symmetry breaking interface.

The spin-orbit coupling on the Ti atoms is described by 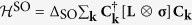, where the angular momentum operator **L** for *l* = 2 is represented in the {*d*_*xz*_, *d*_*yz*_, *d*_*xy*_} basis^33^. This term intermixes the *t*_2*g*_ orbitals and generates three doublets; the upper two doublets are split by 2Δ_SO_ (see Fig. 6). In addition, the deformation of the *t*_2*g*_ orbitals due to the interface potential leads to a hybridization of the *d*_*xz*_, *d*_*yz*_ orbitals with the *d*_*xy*_ orbital, parameterized by


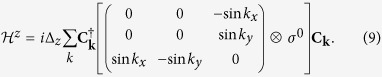


The **k**-dependence in 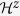 splits the three otherwise doubly degenerate doublets. For small momenta *k*_*x*_ and *k*_*y*_, this splitting acts on the lowest and the highest doublet exactly like a Rashba term in a one-band model. This source of an effective Rashba-like band splitting can be several orders of magnitude larger than the splitting through the relativistic term and thereby is able to explain qualitatively the spin splitting observed at the LAO-STO interface^36^,^37^. Further, if the Fermi energy is tuned to Δ_0_ + Δ_SO_ by an external gate voltage^36^, 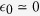 is fulfilled for the highest doublet (see Fig. 6). The SC state can be stabilized by the two lower doublets, whereas the highest generates a non-trivial topological number *C* = ±1.

This three-band model is likely the minimal model which fulfils the requirements discussed above for the realization of topological *s*-wave superconductivity in a solid. Various interface systems with a similar setup are conceivable, however, the formation of a topological SC state is viable only in a restricted parameter range: The Rashba-like band splitting, which is controlled by the parameter *α* in the one-band model, is replaced in the three-band model by *α*_R_ = 2Δ_SO_Δ_*z*_/Δ_0_^34^. Although the magnetic field 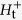, above which *C* ≠ 0 is possible, varies little with *α*_R_, the magnetic field range 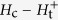 vanishes when *α*_R_ approaches zero. To ensure a wide magnetic field range for the topological state, the parameter *α*_R_ should therefore be larger than the Zeeman splitting and thus also larger than the SC energy gap.

In the following we estimate that the above criteria are indeed satisfied in the candidate system LAO-STO. The interface superconducts below a critical temperature of about 300 mK 38,39 and exhibits an energy gap Δ of about 40 μeV with most likely *s*-wave symmetry^40^. The Rashba parameter *α*_R_ was experimentally estimated to be in the range 20–100 meV^36^, which is compatible with *α*_R_ determined from the three-band model using Δ_0_, Δ_SO_, and Δ_*z*_ from the band-structure calculations of ref. 34. Assuming that 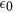 can be adjusted to zero by a suitable gate voltage, the necessary Zeeman splitting *μ*_B_|**H**| > Δ is far smaller than *α*_R_ and corresponds to a magnetic field *H*_t_ ≈ 600 mT (the in-plane *H*_t_ might be somewhat larger). While the measured out-of-plane critical field is *H*_c_(0) ≈ 200 mT^38^ and therefore smaller than *H*_t_, the observed in-plane critical field is 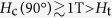
41.

The other important parameter defining *H*_t_ is 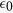, which is controlled by the electron density *n* at the interface. The electron density can be tuned between 1 × 10^−13^ cm^−1^ and 6 × 10^−13^ cm^−1^ 35,39,40. Using Δ_0_ ≈ 50 meV as in ref. 33 and Δ_SO_ ≈ 20 meV^34^, we find that setting 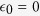, i.e. *E*_F_ = Δ_0_ + Δ_SO_ (see Fig. 6), requires 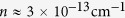. This lies well within reach by a gate voltage. A precise prediction for the electron density at which the topological state should first develop is however difficult, since the value of Δ_0_ and the position of the higher bands is under debate. Density-functional calculations provide rather a value Δ_0_ ≈ 250 meV^34^. However, to account for the low electron densities measured experimentally, the *d*_*xy*_ electrons are likely to be localized in this scenario. The Fermi energy is in this case measured relative to the lower edge of the *d*_*xz*_, *d*_*yz*_ orbitals, i.e., 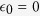 for *E*_F_ = 2Δ_SO_. This is realizable as well within the range of charge densities tunable through a gate voltage.

The verification of a parameter regime which allows for a topological superconducting state could come from measuring the Knight shift in the nuclear magnetic resonance (NMR) frequency, e.g. of the La-nuclei in the first LAO layer. Due to the band splitting generated through the Rashba-type SOC, the superconductor magnetizes in a magnetic field, with a spin susceptibility *χ*_S_ remaining finite down to *T* = 0. Therefore, if a Rashba SOC with strength *α*_R_ ≫ Δ is indeed present at the LAO-STO interface, the drop of the Knight shift at *T*_c_ must be far smaller than expected for a standard *s*-wave superconductor. Further, a kink in *χ*_S_ upon changing the gate voltage might reveal the voltage at which the highest doublet starts to get occupied and the search for topological edge states is most promising.

## Additional Information

**How to cite this article**: Loder, F. *et al*. Route to Topological Superconductivity via Magnetic Field Rotation. *Sci. Rep*. **5**, 15302; doi: 10.1038/srep15302 (2015).

## Supplementary Material

Supplementary Information
