# Supplementary material for: Route to Topological Superconductivity via Magnetic Field Rotation
Source: Sci Rep. 2015 Oct 19;5:15302. doi: 10.1038/srep15302 (PMC4609968; doi:10.1038/srep15302)
Supplement: Supplementary Information [file srep15302-s1.pdf]

Supplementary Informations to "Route to Topological Superconductivity via Magnetic Field Rotation"

Florian Loder[1,2]*, Arno P. Kampf[2], and Thilo Kopp[1]

Center for Electronic Correlations and Magnetism,

[1]*Experimental Physics VI,* [2]*Theoretical Physics III,*

Institute of Physics, University of Augsburg, 86135 Augsburg, Germany

March 27, 2015

In this Supplementary Information we provide a more detailed discussion of certain aspects of superconductivity with Rashba spin-orbit coupling (SOC) in a magnetic field, which are relevant for the structure of the superconducting energy gap.

A Order Parameter in spin- and band-space

Of particular interest is the relation of the spin-singlet order parameter Δ to the intra- and inter-band pairing amplitudes in terms of the eigenstate operators $a_{\mathbf{k}\pm}$ of the SOC split bands. In spin space, the spin-singlet order parameter is represented, as in Eq. (5) of the main text, by the 2×2 matrix

$$\hat{\Delta} = i\sigma^y \Delta = \begin{pmatrix} 0 & \Delta \\ -\Delta & 0 \end{pmatrix} \tag{1}$$

where

$$\Delta = \frac{V}{2N} \sum_{\mathbf{k}} \langle c_{\mathbf{k},\downarrow} c_{-\mathbf{k},\uparrow} - c_{\mathbf{k},\uparrow} c_{-\mathbf{k},\downarrow} \rangle. \tag{2}$$

*Correspondence to florian.loder@physik.uni-augsburg.de

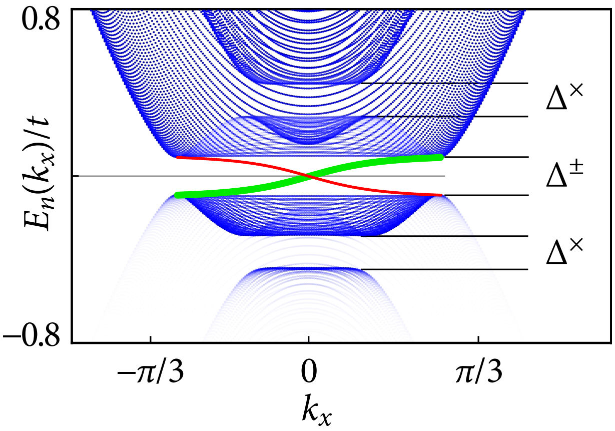

Supp. Fig. 1: Illustration of the energy gaps induced by the different pairing contributions of an s-wave superconductor with an out-of-plane magnetic field $H_z > H_\mathrm{t}$ (with parameters as in Fig. 5 of the main text). The energy gap centered around $E = 0$ originated from the intra-band pairing term $\Delta^+ = \Delta^-$, whereas the inter-band pairing term $\Delta^\times$ generates two interior gaps above and below the Fermi energy, where the normal-state bands $\xi^+_\mathbf{k}$ and $-\xi^-_{-\mathbf{k}}$, or similarly $\xi^-_\mathbf{k}$ and $-\xi^+_{-\mathbf{k}}$, cross. The green (red) lines mark the in-pay modes originating from the upper (lower) edge.

In this section we focus exclusively on pairing with zero center-of-mass momentum (COMM), although the following derivations are valid for arbitrary magnetic field directions. Finite COMM as required in the case of a sufficiently large in-plane component of the magnetic field (see Sec. B) is implemented simply by replacing $-\mathbf{k}$ by $-\mathbf{k}+\mathbf{q}$.

In the presence of SOC, the operator $c_{\mathbf{k}s}$ no longer annihilates an eigenstate of the normal state Hamiltonian. Instead, the band operators $a_{\mathbf{k}\pm}$ refering to the eigenstates with energies $\xi^\pm_\mathbf{k}$ are obtained by the transformation

$$\begin{pmatrix} a_{\mathbf{k}+} \\ a_{\mathbf{k}-} \end{pmatrix} = \sqrt{\frac{h_{\mathbf{k},x} - ih_{\mathbf{k},y}}{2h_\mathbf{k}}} \begin{pmatrix} 1 & \phi^+_\mathbf{k} \\ 1 & \phi^-_\mathbf{k} \end{pmatrix} \begin{pmatrix} c_{\mathbf{k},\uparrow} \\ c_{\mathbf{k},\downarrow} \end{pmatrix} \tag{3}$$

with

$$\phi^\pm_\mathbf{k} = \frac{h_{\mathbf{k},z} \pm h_\mathbf{k}}{h_{\mathbf{k},x} - ih_{\mathbf{k},y}}, \tag{4}$$

and $h(\mathbf{k}) = |\mathbf{h}(\mathbf{k})|$ is the absolute value of the Bloch vector $\mathbf{h}(\mathbf{k})$. Applying this transformation on $\hat{\Delta}$ generates an order parameter $\tilde{\Delta}$ for pairing the normal state eigenstates with energies $\xi^\pm_\mathbf{k}$:

$$\begin{aligned} \tilde{\Delta}_\mathbf{k} &= \begin{pmatrix} \Delta^+_\mathbf{k} & \Delta^\times_\mathbf{k} \\ -\Delta^\times_\mathbf{k} & \Delta^-_\mathbf{k} \end{pmatrix} \\ &= \frac{\sqrt{h^2_{\mathbf{k},x} + h^2_{\mathbf{k},y}}}{2h_\mathbf{k}} \begin{pmatrix} -(\phi^+_\mathbf{k} - \phi^+_{-\mathbf{k}})\Delta & -(\phi^+_\mathbf{k} - \phi^-_{-\mathbf{k}})\Delta \\ (\phi^+_\mathbf{k} - \phi^-_{-\mathbf{k}})\Delta & -(\phi^-_\mathbf{k} - \phi^-_{-\mathbf{k}})\Delta \end{pmatrix}. \end{aligned} \tag{5}$$

Here, $\Delta^{+}_{\mathbf{k}} = -\Delta^{-}_{\mathbf{k}}$ represents the intra-band pairing gaps, whereas $\Delta^{\times}_{\mathbf{k}}$ represents the inter-band pairing gap. In zero magnetic field, $\phi^{+}_{\mathbf{k}} - \phi^{-}_{-\mathbf{k}} = 0$ and therefore only intra-band pairing occurs. However, in finite magnetic fields there is always a finite inter-band order parameter $\Delta^{\times}_{\mathbf{k}}$. This term opens a gap at the intersections of the dispersions $\xi^{+}_{\mathbf{k}}$ and $-\xi^{-}_{-\mathbf{k}}$, or $\xi^{-}_{\mathbf{k}}$ and $-\xi^{+}_{-\mathbf{k}}$, which are above and below the Fermi energy, respectively (illustrated in Supp. Fig. 1). These interior gaps are enforced by the assumed contact pairing interaction, which allows exclusively for s-wave singlet pairing. Inter-band pairing and interior gaps are likely absent for spin-triplet pairing. Note that for an out-of-plane magnetic field, the absolute values $|\Delta^{\pm}_{\mathbf{k}}| = \Delta^{\pm}$ and $|\Delta^{\times}_{\mathbf{k}}| = \Delta^{\times}$ are $\mathbf{k}$-independent. In the presence of an in-plane field component, also the absolute values are $\mathbf{k}$-dependent.

If $\mathbf{H}$ is orientated strictly in-plane (here we assume $\mathbf{H} = \mathbf{H}_{\parallel} = (0, H_y, 0)$), then

$$\phi^{+}_{\mathbf{k}} = -\phi^{-}_{\mathbf{k}} = \frac{h_{\mathbf{k},x} - i h_{\mathbf{k},y}}{h_{\mathbf{k}}}. \tag{6}$$

It follows that in this case,

$$\phi^{\pm}_{\mathbf{k}} - \phi^{\pm}_{-\mathbf{k}} = \begin{cases} 1, & \alpha|\sin k_x| > \mu_{\mathrm{B}} H_y \\ 0, & \alpha|\sin k_x| < \mu_{\mathrm{B}} H_y \end{cases} \tag{7}$$

for all $\mathbf{k}$ on the k_x-axis. Since in the regime $H_y > H_{\mathrm{t}}$, $\mu_{\mathrm{B}} H_y > \alpha|\sin k_{\mathrm{F},x}|$ is always fulfilled, the intra-band gaps $\Delta^{\pm}_{\mathbf{k}}$ close on the two Fermi points $k^{\pm}_{\mathrm{F},x}$ on the positive and the negative k_x-axis, respectively, for $H_y > H_{\mathrm{t}}$ [see Supp. Fig. 2 (a)]. This gap closing demonstrates the impossibility to form spin-singlet pairs in the intra-band channel on the k_x-axis in the regime $\alpha|\sin k_y| < \mu_{\mathrm{B}} H_y$ (cf. the spin configuration on the k_x-axis in Fig. 1 (c)).

B In-plane magnetic fields and finite momentum pairing

In this section we illustrate the necessity of pairing with a finite COMM $\mathbf{q}$ in the simultaneous presence of a Rashba SOC and an in-plane magnetic field. Supp. Fig. 2 (a) is identical to Fig. 5 (c) of the main text, showing the energy spectrum in a stripe geometry for an in-plane magnetic field $H_y > H_{\mathrm{t}}$. Since in the normal state of this "situation (A)" only the $\xi^{-}_{\mathbf{k}}$-band is partially occupied, naturally only intra-band pairing occurs, with a COMM $\mathbf{q}^{-}$. Because the intra-band energy gap appears at the crossings of the two bands $\xi^{-}_{\mathbf{k}}$ and $-\xi^{-}_{-\mathbf{k}+\mathbf{q}^{-}}$, the ideal situation for pairing would be the existence of a $\mathbf{q}^{-}$ for which $\xi^{\pm}_{\mathbf{k}} = \xi^{\pm}_{-\mathbf{k}+\mathbf{q}^{-}}$ for all $\mathbf{k}$. This condition cannot be fulfilled exactly for a tight-binding description (since the in-plane magnetic field slightly deforms the band structure). Nevertheless it is sufficient for the formation of electron pairs, if $\xi^{\pm}_{\mathbf{k}} - \xi^{\pm}_{-\mathbf{k}+\mathbf{q}^{-}} < \Delta$ for all $\mathbf{k}$ on the respective Fermi surface. The COMM $\mathbf{q}^{-}$ chosen to fulfil this latter condition ensures that the indirect energy gap does not close [cf. Supp. Fig. 2 (a)], except at the two $\mathbf{k}$-points described in the main text.

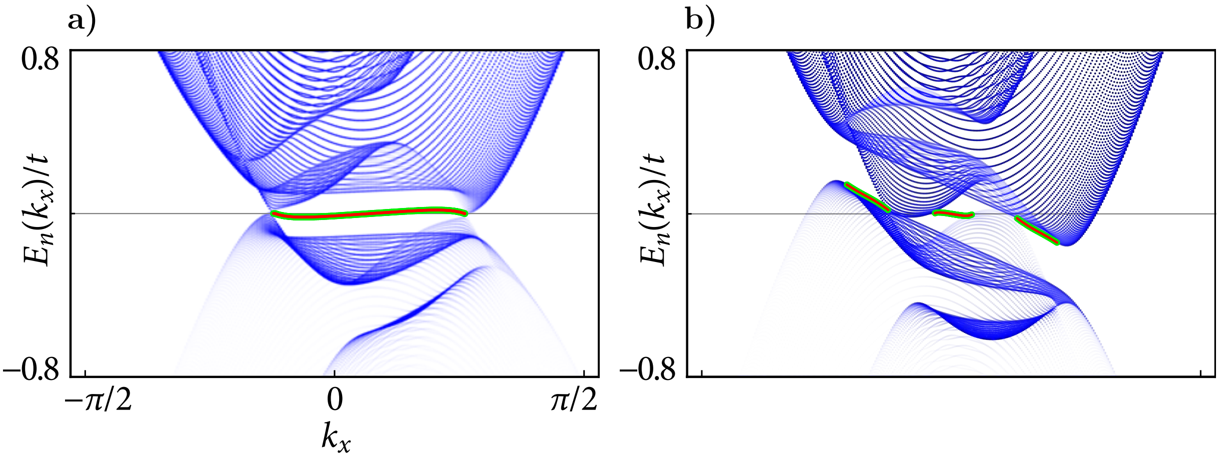

Supp. Fig. 2: Illustration of the effect of a finite COMM on the energy gap of an s-wave superconductor in an in-plane magnetic field. The asymmetry $\xi^{\pm}_{\mathbf{k}} \neq \xi^{\pm}_{-\mathbf{k}}$ in the simultaneous presence of an in-plane magnetic field and Rashba SOC leads to tilted edges of the $\xi^{-}_{\mathbf{k}}$-continuum above and below the energy gap (b). This tilting can be corrected by choosing an appropriate COMM $\mathbf{q}^{-}$ which compensates the shift of the energy bands. (a) redisplays Fig. 5 (c) in the main text, whereas in (b), Δ is fixed to the same value as in (a), but with $\mathbf{q}^{-} = 0$. In (b), two degenerate edge modes are present as well, although they merge with the continuum around the two direct-gap closing points.

In fact, the free energy is minimized by the COMM $\mathbf{q}^{-} = (q, 0)$ [for $\mathbf{H}_{\parallel} = (0, H_y, 0)$] with the smallest q for which the energy gap does not close indirectly. This condition translates into the requirement that the energy difference

$$\delta\xi(q) = \xi^{-}_{\mathbf{k}^{+}_{\mathrm{F}}} - \xi^{-}_{-\mathbf{k}^{+}_{\mathrm{F}}+\mathbf{q}^{-}} \tag{8}$$

must be smaller than the energy gap around the Fermi energy. Here, $\mathbf{k}^{+}_{\mathrm{F}}$ is the Fermi momentum on the positive k_x axis. If the magnetic field orientation is not close to in-plane, i.e., $H_y < \alpha \sin k^{\pm}_{\mathrm{F},x}$, the energy gap is simply given by Δ. However, upon approaching an in-plane orientation, the energy gap closes directly at the two Fermi points on the k_x-axis. Therefore, q is fixed by the condition $\delta\xi(q) = 0$ for an in-plane magnetic field.

Supplementary Fig. 2 (b) shows the energy spectrum of the same superconducting system as Fig. 2 (a), but setting q ad hoc to zero. The energy gap in this case is obviously indirectly closed. Additionally, two direct closing points exist at momenta $|k^{\pm}_{x}| < |k^{\pm}_{\mathrm{F},x}|$. It is important to notice that the self-consistent solution for Δ vanishes abruptly, if the energy gap closes indirectly upon increasing $\mathbf{H}$. In Supp. Fig. 2 (b), Δ is kept fixed for illustration purposes, although no self-consistent solution of this kind exists.

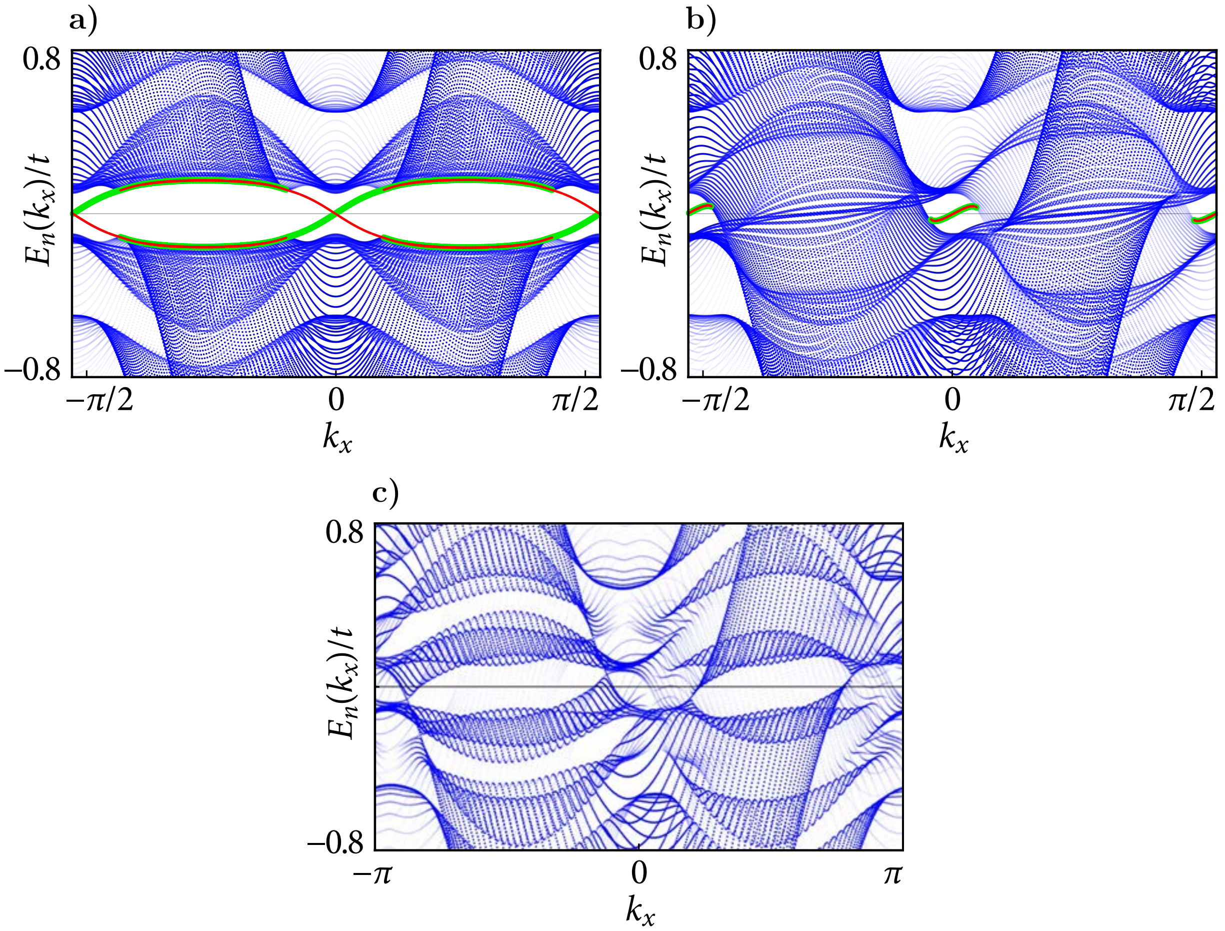

Supp. Fig. 3: Energy spectrum of the topologically non-trivial state $\mu_B|\mathbf{H}| > \sqrt{(\epsilon_0 + 4t)^2 + \Delta^2}$ for "situation B" with $\epsilon_0 + 4t = \mu = 0$, $\mu_B|\mathbf{H}| = 0.3\,t$ and (a) an out-of-plane magnetic field, (b) an in-plane magnetic field with zero COMM and (c) an in-plane magnetic field with COMMs $\mathbf{q}^+$ and $\mathbf{q}^-$ optimizing pairing in the $\xi^+_{\mathbf{k}}$- and the $\xi^-_{\mathbf{k}}$-band. In (a), there is a full energy gap with topological edge states crossing each other twice, i.e., $C = 2$. While no energy gap around the Fermi energy remains in (b), finite COMM pairing can only partially recover the energy gap. The spectra in (a) and (b) are calculated for a stripe geometry. The spectrum in (c) is calculated using periodic boundary conditions, therefore edge states are absent.

C Topology in *s*-wave superconductors close to half filling

In the main text we argued that in the density regime close to half filling ($|\mu| < 2t$, entitled "situation (B)"), no integer topological number C can be defined, because finite COMM pairing with $\mathbf{q}^+$ and $\mathbf{q}^-$ necessarily generates in-gap states. This phenomenon is specifically illustrated here in Supp. Fig. 3: For an out-of-plane magnetic field, the topological state for $\mu_B H_z > \sqrt{(\epsilon_0 + 4t)^2 + \Delta^2}$ has $C = 2$ [3]. Consequently, the emerging edge states cross each other twice, at $k_x = 0$ and at $k_x = \pi$ [Supp. Fig. 3 (a)]. Such a

topological state is possible for field orientations forming a small angle with the z-axis. Upon rotating the magnetic field into the plane, but keeping $\mathbf{q}^{\pm} = \mathbf{0}$, the edge states become degenerate but remain well defined in those sections of k_x where a direct energy gap persists. [Supp. Fig. 3 (b)]. However, the shift of the two Fermi surfaces in opposite directions leads to a closing of the energy gap over an extended region in momentum space. As discussed above, such a situation with $\mu_{\mathrm{B}} H_y > \sqrt{(\epsilon_0 + 4t)^2 + \Delta^2}$ but $\mathbf{q} = \mathbf{0}$ does not correspond to a state of minimum free energy, but serves only for demonstrating the effect of the in-plane magnetic field.

Unlike in the low-density regime ($\mu < -2t$, "situation (A)"), it is here not possible to remove the in-gap states by compensating the shift of the Fermi surfaces with finite COMMs. Since both, the $\xi^{+}_{\mathbf{k}}$- and the $\xi^{-}_{\mathbf{k}}$-band, are partially occupied, two order parameters $\Delta_{\mathbf{q}^+}$ and $\Delta_{\mathbf{q}^-}$ are required, where $\mathbf{q}^+ \approx -\mathbf{q}^-$. The corresponding order parameter in real space exhibits a stripe-like pair-density-wave modulation of the form $\cos^2(q^+_x - q^-_x)$, which has lines of zero pair density [1,4,5]. This implies that the density of states of such a superconductor does not vanish at the Fermi energy, i.e., in-gap states necessarily remain.

The energy spectrum of this superconducting state is shown in Supp. Fig. 3 (c), where $\mathbf{q}^+$ and $\mathbf{q}^-$ are chosen to compensate the shift of the respective Fermi surfaces ideally. Unlike in Supp. Figs. 3 (a) and (b), this spectrum is calculated using periodic boundary conditions, since the assignment of the COMMs $\mathbf{q}^+$ and $\mathbf{q}^-$ to the correct pairing terms in real space is numerically difficult. Therefore edge states are absent.

References

[1] F. Loder, A. P. Kampf, and T. Kopp, Phys. Rev. B **81**, 020511(R) (2010).

[2] F. Loder, A. P. Kampf, and T. Kopp, J. Phys.: Condens. Matter **25**, 362201 (2013).

[3] M. Sato and S. Fujimoto, Phys. Rev. B **79**, 094504 (2009).

[4] D. F. Agterberg and H. Tsunetsugu, Nature Phys. **4**, 639 (2008).

[5] E. Berg, E. Fradkin, S. A. Kivelson, and J, M. Tranquada, New J. Phys. **11**, 115004 (2009).
